# Dietary Intakes of Long-Chain Polyunsaturated Fatty Acids and Impulsivity: Comparing Non-Restricted, Vegetarian, and Vegan Diets

**DOI:** 10.3390/nu16060875

**Published:** 2024-03-18

**Authors:** Mitchell K. Byrne, Rebecca Cook, Janina C. D. Murta, Daniel Bressington, Barbara J. Meyer

**Affiliations:** 1 Faculty of Health, Charles Darwin University, Ellengowan Drive, Darwin, NT 0810, Australia; mitchell.byrne@cdu.edu.au (M.K.B.); beckalpha74@icloud.com (R.C.); janina.catalaodionisiomurta@cdu.edu.au (J.C.D.M.); daniel.bressington@cdu.edu.au (D.B.); 2 Faculty of Nursing, Chiang Mai University, 110/406 Inthawaroros Road, Sri Phum District, Chiang Mai 50200, Thailand; 3 School of Medical, Indigenous and Health Sciences, Lipid Research Centre, Molecular Horizons, University of Wollongong, Wollongong, NSW 2522, Australia; 4 Illawarra Health and Medical Research Institute, Wollongong, NSW 2522, Australia

**Keywords:** omega-3 long-chain polyunsaturated fatty acids, food frequency questionnaire, Barrat impulsiveness scale, trait impulsivity, attentional

## Abstract

Background: Research suggests a link between deficiencies in omega-3 long-chain polyunsaturated fatty acids (LCPUFAs) and impulsivity among psychiatric populations. However, this association is less evident in non-clinical populations. As omega-3 LCPUFAs are predominantly sourced through fish consumption, non-fish dieters may be more vulnerable to higher impulsivity. Methods: This cross-sectional observational study explored the association between lower intakes of omega-3 LCPUFA food sources and higher self-reported measures of impulsivity among healthy adults consuming non-restricted, vegetarian, and vegan diets. Results: The results from the validated Food Frequency Questionnaire showed significantly lower estimated omega-3 LCPUFA intakes among vegans and vegetarians when compared with people consuming non-restricted diets. Furthermore, although all groups scored within the normal range of impulsivity measures, vegans scored comparatively higher. Vegans also scored significantly higher in impulsivity control relating to attention than those consuming non-restricted diets. Conclusions: The significantly lower omega-3 LCPUFA dietary intakes in the vegan diets were associated with higher scores in the second-order attentional aspect of self-reported impulsiveness.

## 1. Introduction

There is growing recognition of the relationship between diet and mental health, particularly the beneficial role of omega-3 LCPUFAs in improving the main symptom dimensions in psychiatric populations, including impulsivity. Impulsivity is arguably associated with the most significant number of mental health conditions in clinical and non-clinical populations [1]. According to the fifth edition of the American Psychiatric Association’s (APA) Diagnostic and Statistical Manual of Mental Disorders (DSM-5), the disinhibitory trait of impulsivity is characteristic of 18 disorders, including attention deficit hyperactivity disorder (ADHD), autism spectrum disorder (ASD), and bipolar disorder (BPD) [2].

In clinical and non-clinical populations, impulsivity can present in various forms, including attention deficit, poor concentration, high risk-taking, poor decision-making, and unplanned or socially unfavourable reactive behaviour [3]. These behavioural and cognitive presentations impair everyday functioning and can have adverse social consequences regarding education, employment, and personal relationships [1]. Thus, impulsivity is an important target for intervention [3].

Despite progress in the development of pharmacological treatments to help manage symptoms of clinical impulsiveness, some studies suggest that they may be unfavourable due to adverse effects, including appetite loss, insomnia, abdominal pains, long-term safety concerns and unsuitability for use in sub-clinical populations [4,5]. At the same time, there is growing interest in non-pharmacological approaches to address impulsive behaviours. Omega-3 LCPUFAs, which are widely studied as important nutrients for brain function and supplementation in mental health disorders [6], are now being widely tested as potential nutraceuticals for improving impulsive behaviour [7].

This growing research interest builds on previous evidence indicating a relationship between omega-3 LCPUFA deficiency and impulsivity-related issues including aggression, hostility [8,9], poorer cognitive performance [10,11,12], psychopathologies [6,13,14,15], and self-regulation disorders such as attention deficit hyperactivity disorder (ADHD), autism spectrum disorder (ASD), bipolar disorder (BPD), and anti-social personality disorder and aggression [8,9,16,17]. It also builds on research suggesting that the core executive function of impulse control requires the efficient functioning of the prefrontal cortex [18], which in turn requires an adequate balance of omega-3 LCPUFAs and omega-6 LCPUFAs for optimal operation [19]. These two families of LCPUFAs include omega-3 eicosapentaenoic acid (EPA), docosapentaenoic acid (DPA), docosahexaenoic acid (DHA) and its precursor, alpha-linolenic acid (ALA), and omega-6 arachidonic acid (AA) and its precursor linoleic acid (LA) [20].

Studies involving nutrition and mental health consistently indicate that while the optimal balance of *n*-3 and *n*-6 is 1:1, the globally predominant Western diet (with a high energy content and low nutritional value) is misaligned with our genetic disposition, at a balance of approximately 1:16–20 [21,22,23]. Additionally, although preferential diets such as vegan and vegetarian are healthy in that they are lower in total fats, cholesterol, and saturated fats, they tend to show greater deficiencies of *n*-3 and higher concentrations of *n*-6 when compared to non-restricted diets [24]. The exclusion of fish may result in *n*-3 LCPUFA deficiencies and a predisposition toward greater impulsivity [24].

There is emerging evidence showing that omega-3 LCPUFA supplementation can have a beneficial effect in addressing impulsivity [15]. However, research regarding the benefits of omega-3 LCPUFAs for the management of mental health conditions in the general population is less widely explored [10].

This study aimed to investigate the unexplored association between the dietary intake of omega-3 LCPUFAs in restricted diets and the levels of trait impulsivity using a cross-sectional and quantitative approach among the general adult population. It was hypothesised that participants following vegan and vegetarian diets would show lower estimated omega-3 LCPUFA dietary intakes than the non-restricted dietary group. It was also hypothesised that participants following vegan and vegetarian diets would score higher in the self-reported measures of impulsivity compared to the non-restricted diet group.

## 2. Materials and Methods

### 2.1. Study Design

The current cross-sectional observational study used a between-subject design for the comparison of means (or median for non-normally distributed data) [25]. The independent variable was the usual diet type, consisting of three groups: non-restricted, vegetarian, and vegan. The dependent variables measured included total and breakdown estimates of omega-3 (ALA, EPA, DPA and DHA), and omega-6 (LA and AA), and a self-reported measure of trait impulsivity, yielding a total and sub score of three second-order factors (attentional, motor, and non-planning).

### 2.2. Participants

This study was approved by the Charles Darwin University Human Research Ethics Committee (H21040). To determine group differences based on dietary omega-3 estimates in impulsivity scores, an a priori power analysis for the primary outcome using G*Power [26] was conducted with input parameters indicating the power (1 − β) set at 0.95, the effect size *f* set at 0.25, and the err probability α set at 0.05 for three groups. The output generated indicated that the total sample size would need to be N = 252 for group differences to reach statistical significance at the 0.05 level.

A total of 695 healthy Australian adult volunteers aged 18 years and over responded to an online invitation via Qualtrics to participate in an anonymous survey investigating diet and self-regulation. All data were collected and stored following corresponding research management procedures, including obligations under the 2018 National Statement on Ethical Conduct in Human Research. No incentive to participate was offered.

The initial sampling method [27] began as non-target-specific, belonging to the convenience/snowball type, with volunteers invited to share the survey invitation link. However, a significant difference in group sizes became apparent early in the recruitment stage. Ethics approval was sought and granted to amend the sampling method from random to stratified, targeting vegan and vegetarian interest groups to balance group sizes.

### 2.3. Questionnaires

The Qualtrics online survey platform was the preferred option to achieve optimal data for analysis, providing both response quality and expert review functions, including Internet BOT (robot) detection (i.e., a software application to imitate human internet activity), fraudulent/duplicate responding, and poor completion (https://www.qualtrics.com/) (accessed on 22 July 2022).

The polyunsaturated fatty acid food frequency questionnaire (PUFA FFQ) is an expanded electronic version of the original and the first of its kind, developed in 2008 by Sullivan and colleagues [28,29], providing a comprehensive list of Australian foods containing LCPUFAs [30]. The results from the PUFA FFQ were calculated automatically, providing quantitative total amounts (expressed as grams per 100 g of edible portion) for ALA, EPA, DPA and DHA, and omega-6 LA and AA.

To determine “true intake”, the triad method was used, which is a statistical model which uses a triangular comparison between the questionnaire, the reference method (3-day weighed food records) and a biomarker (in this case [30], both plasma and erythrocytes were used as biomarkers). The closer the validity coefficient is to 1, the closer the intake estimated by the dietary assessment is to the “true intake”. The validity coefficient for the “true intake” of the PUFA FFQ was 0.78 and the validity coefficient for the “true intake” of the erythrocyte omega-3 LCPUFA was 0.79. Furthermore, reproducibility was assessed 2 to 4 months after the first PUFA FFQ and the second PUFA FFQ and the Spearman correlation coefficient was 0.85, showing that the PUFA FFQ was reproducible. Moreover, the study participants in these validation studies [28,29,30] consumed varied diets, including vegan, vegetarian and non-restricted diets, hence the PUFA FFQ was validated for use with people consuming these diets. 

Furthermore, this PUFA FFQ was compared to a generic FFQ using the triad method and the validity coefficient of the “true intake” of the PUFA FFQ was 0.78 compared to the generic FFQ validity coefficient of 0.23 [31], demonstrating that the PUFA FFQ is superior to generic FFQ to determine omega-3 LCPUFAs. 

Therefore, the PUFA FFQ is reproducible and has been validated for blood omega-3 LCPUFAs, and hence it can be used in research without the need to take blood samples to measure dietary omega-3 LCPUFA intake [28,29,30].

The 30-item self-report Barratt Impulsiveness Scale (BIS-11), one of the most common measures used in mental health research, was developed to measure the disinhibitory trait of impulsivity and shows both high internal consistency α = 0.79−0.83 and high reproducibility *r* = 0.85 [32,33]. When conducted online, self-report measures of impulsivity have been shown to be more strongly related to real-world overt behaviour than unnaturalistic behavioural testing procedures, so they were deemed suitable for the current study [34].

Furthermore, the BIS-11 incorporates six first-, and three second-order subscale factors incorporating both personality and behavioural aspects in order to determine the contribution of each toward the overall level of impulsiveness [32]. The first-order factors include attention, cognitive instability, motor, perseverance, cognitive complexity, and self-control. The second-order factors include attentional, motor, and non-planning. A detailed description of the BIS-11 items and factor loadings is provided in Appendix A Table A1.

Items on the BIS-11 are scored on a 4-point Likert scale: Rarely/Never = 1; Occasionally = 2; Often = 3; and Almost Always/Always = 4. Scores were calculated as the sum of responses for all 30 items, resulting in a raw total between 30 to 120. Previously, a total score of 74, one standard deviation above the mean reported by Patton et al. [32], was used to label someone as impulsive [32,33]. Stanford et al. [33] has since concluded that a total score of 72 or more is troublesome and symptomatic of high impulsivity. The current study used the same cut-off points for scoring as suggested by Stanford et al. [33]: highly impulsive—higher than or equal to 72; within the normal range—between 52 and 71; and low in impulsiveness or highly controlled—lower than or equal to 51 [33]. Although most studies only calculate total BIS-11 scores, because of the multidimensional nature of trait impulsivity, Stanford et al. [33] advised that second-order factors should be analysed and interpreted separately for a more accurate measure of participants’ overall level of impulsiveness. Used successfully in prior research [35], the method used to derive cut-off scores for BIS-11 second-order factors was to sum all item responses relating specifically to each of the three sub-dimensions, with higher scores representing higher impulsiveness: attentional, motor, and non-planning were scored from possible totals of 32, 44, and 44, respectively.

### 2.4. Recruitment and Data Collection

Participant data were collected by distributing a Qualtrics survey platform link via posts to non-diet-specific online communities and interest groups relating to food, diet, lifestyle, and recipe swapping. Stratified targeting of specific groups of vegans and vegetarians was later included in an attempt to equalise group numbers. Upon following the survey link, participants were provided information regarding the average time to complete, anonymity assurance, the right to withdraw, contact details for the principal investigator, the corresponding research integrity and ethics team, and the research topic (diet and self-regulation). The hypotheses were not disclosed to avoid biased, defensive, or planned participant responses [36]. Informed consent was indicated via a forced response function within the survey information page, prompting a binary response: ‘*I consent/I do not consent*’.

In the case of a non-consensual response, participants could still explore elements within the survey content, increasing the potential for obtaining reconsidered consent. In either case, the data exported from Qualtrics flagged non-consensual responses for easy identification and subsequent removal from the participant pool. No respondent IP addresses, location data, personal or identifying information such as names, phone numbers, residential or postal addresses were collected.

For the BIS-11 component, patients were encouraged to answer quickly and honestly each of the 30 questions relating to attentional (Att2nd; *I often have extraneous thoughts when thinking*), motor (Mot2nd; *I do things without thinking*), or non-planning (NonPl2nd; *I am more interested in the present than the future*) second-order factors. An external link provided within the Qualtrics platform enabled access to the second PUFA FFQ component. For the PUFA FFQ component, participants were instructed to indicate their estimated dietary intakes of various foods listed, including portion size and frequency of consumption. Comprising the first three digits of their unique GroupID (later used to pair BIS-11 and PUFA FFQ responses appropriately), participants were instructed to identify their diet preference as either NR = non-restricted, VTN = vegetarian, or VGN = vegan. Upon completing the second PUFA FFQ component, participants provided a one-page summary of their estimated total omega-3 consumption (expressed as mg) per day.

Although ethical risk was deemed negligible regarding participants following vegan and vegetarian diets, content disclosure was provided regarding questions relating to meat products. This transparency was necessary because many individuals who follow restrictive diets are guided by deeply held morals more than health concerns or perceived benefits [37]. The option was provided to either skip these questions or discontinue participation. However, these questions were retained for those on unrestricted diets given the observations that meat contains both omega-6 and omega-3 [38].

### 2.5. Statistical Analysis

The following analyses, using SPSS 28 [39], were conducted to investigate the possible association between estimated dietary intakes of omega-3 and self-reported measures of trait impulsiveness between three dietary preferences. The four ANOVAs conducted for normally distributed data were related to BIS scores. The between-subject variable was dietary preference: non-restricted (NR), vegetarian (VTN), and vegan (VGN). Dependent variables analysed for interpretation included the BIS total raw scores and BIS second-order factors: Att2nd, Mot2nd, and Nonpl2nd. A further four non-parametric analyses followed using the Kruskal–Wallis test. LCPUFA-dependent variables included omega-3 total combined (EPA, DPA, DHA), omega-3 EPA, omega-3 DHA, and omega-6 total (LA, AA).

## 3. Results

The participants that completed both the PUFA FFQ and the BIS (*n* = 333) consisted of non-restricted (NR; *n* = 212; 63.7%), vegetarian (VTN; *n* = 45; 13.5%), and vegan (VGN; *n* = 76; 22.8%). Females (*n* = 302) significantly outnumbered males (*n* = 31) among the participants (90.7%). Ages ranged from 19 to 65 years, and the majority were between 45 and 49 years of age (14.4%). Both the mean and median age groups fell between 40 and 44 years of age. Additional descriptive data are reported as mean ± standard deviation, while non-parametric test results are reported using median values. No exploration was conducted between gender groups for either FFQ intake estimates or BIS scores, as the gender sample size difference was too great for any meaningful comparison.

Before analysis, visual inspection of histograms, normal Q-Q plots, and box plots indicated heavy outliers in the data and extreme positive skewness across groups for FFQ (totals and separate omega-3 and omega-6) intake estimates. Attempted transformations using Log10 and SqRt functions were unsuccessful. Calculated measures of skewness and kurtosis for FFQ estimated intakes indicated a violation of normality. Shapiro–Wilk’s test further confirmed a significant departure from normality (*p* < 0.001) for FFQ estimated intakes across all three groups and for estimated intakes of omega-3 LCPUFAs (EPA, DPA, DHA), and omega-6 LCPUFAs (LA, AA) as both separate and combined totals.

The visual inspection of histograms, normal Q-Q plots, and box plots indicated that BIS scores appeared evenly distributed across all groups, apart from the BIS Att2nd scores for NR with skewness of 0.725 (SE = 0.167). However, considering absolute values for skewness, visual inspection, and sample size, this was accepted as normally distributed [40]. Shapiro–Wilk’s test confirmed normalcy for the distribution of total and second-order factor BIS scores across groups [41].

In consideration of unbalanced group sizes, data considered as both normally distributed and meeting the homogeneity assumption were evaluated using a one-way ANOVA where Hochberg’s GT2 served as the post hoc procedure for any significant findings [42]. All non-parametric data were evaluated for stochastic dominance using a Kruskal–Wallis test, followed by Dunn’s (1964) test with a Bonferroni adjusted *p*-value calculated as 0.05/3, resulting in a significance threshold of 0.017 [43]. While the examination of data not specified a priori leads to the risk of type 2 errors, this is usually associated with less conservative probability estimates, such as *p* = 0.05. Therefore, the robust coefficient of *p* < 0.002 attenuates the risks associated with post hoc analysis.

### 3.1. LCPUFA Intakes

The distributions of the omega-3 LCPUFA intakes (and EPA and DHA) were similar for all 3 diet groups, as assessed via the visual inspection of a boxplot. Table 1 shows the results of median LCPUFA intakes.

Total omega-3 LCPUFA (EPA, DPA, DHA) intakes were 14-fold higher (*p* < 0.001) in people consuming the non-restricted diet compared to those consuming vegetarian diet. The EPA intakes were 17.5-fold higher (*p* = 0.001) in those consuming the non-restricted diet compared to those consuming the vegetarian diet and the DHA intakes were 11-fold higher (*p* < 0.001) in those consuming the non-restricted diet compared to those consuming the vegetarian diet.

The total omega-6 (LA and AA) intakes in those consuming the vegan diet were 1.5-fold higher (*p* = 0.002) than those consuming the non-restricted diet and 1.7-fold higher (*p* = 0.002) than those consuming the vegetarian diet.

### 3.2. BIS-11 Scores

Table 2 shows the total BIS-11 and the second-order factors scores from the non-restricted, vegetarian and vegan groups.

Self-reported ratings of impulsivity (BIS-11 total scores) showed no difference between the three groups. The second-order factor self-reported ratings of Att2nd were highest (*p* = 0.002) in the vegan group and approximately 10% higher than the other two diets groups.

## 4. Discussion

This cross-sectional study investigated the association between omega-3 LCPUFA separate and combined total intakes and total and sub-dimensional self-reported measures of trait impulsiveness among 333 healthy adults across three diets: non-restricted, vegetarian, and vegan. The hypotheses were supported by the vegan and vegetarian groups showing significantly lower omega-3 LCPUFA dietary intakes and scoring comparatively higher in self-reported impulsiveness. While group means for both total and second-order factor BIS-11 scores fell within the normal range, evidence was provided of an association between lower dietary intake of omega-3 LCPUFAs (EPA, DPA, DHA) and higher scores on the BIS-11 second-order factor of attention for vegans (η^2^ = 0.036).

Although less proportional in strength, this finding corresponds to existing research demonstrating a negative correlation between omega-3 LCPUFA status and lower attentional inhibition in men [44], women [12] and children [45]. Additionally, using the same measure of impulsiveness (BIS-11), genetic studies have found an association between allelic variations in dopamine D2 receptor genes and attentional impulsiveness [46]. Research has demonstrated that supplementation with omega-3 PUFAs increases brain activation in prefrontal areas associated with attention [47] and has clinically meaningful effects in the management of attention deficit hyperactivity disorder [48]. It is likely that deficits in omega-3 LCPUFA intake associated with dietary choice impairs prefrontal activation; however, further investigation may be warranted to fully understand the role of omega-3 LCPUFAs specific to the attentional inhibition aspects of trait impulsiveness. 

Both AA and DHA are the main PUFAs in the brain and both are required for neurological development as well as throughout the lifespan [22]. It is estimated that 90% of omega-3 PUFAs in the brain are DHA [49]. Humans can synthesise AA from LA; however, the synthesis of DHA from ALA is limited [50]. Certainly, in children with amino acid metabolism disorders who are treated with a protein-restricted diet and hence have an extremely limited consumption of AA, their erythrocyte levels of AA are comparable to healthy children, suggesting that these children can convert LA to AA [51]. However, these same children do not convert ALA to DHA, as their DHA levels are 30% lower compared to healthy children [51]. Therefore, it is no surprise that people consuming a vegan/vegetarian diet have no/little DHA in their diet and subsequently their omega-3 status is low [24]. 

Randomised placebo-controlled trials have shown that omega-3 supplementation can improve memory and reaction time in young healthy adults [52] and benefit mental health in older people with mild cognitive impairment [53], suggesting that omega-3 conserves brain function as humans age. The possible mechanisms for the health benefits of omega-3 include improved neural membrane stability, improved serotonin and dopamine transmission, anti-inflammatory effects and pro-resolving inflammation [54]. 

Impulsivity has been identified as a core feature of attention deficit hyperactivity disorder (ADHD), as noted in the diagnostic criteria outlined in the Diagnostic and Statistical Manual of Mental Disorders, Fifth Edition [2]. Numerous authors have identified an association between ADHD and aggressive behaviour [55,56], prompting research into lifestyle-related preventative interventions, such as dietary management [21,44,57,58].

Additionally, the significant main effects found concerning lower omega-3 LCPUFA intakes among vegans and vegetarians compared with non-restricted diets were consistent with numerous studies, thus demonstrating the need for supplementation in restrictive diets [59,60].

As fish consumption is excluded for those adhering to vegan or vegetarian diets (with the exception of pesco-vegetarians), an alternative source is microalgae and seaweed. However, the levels of EPA and DHA found in these plants are significantly lower than the levels found in fish due to the far lower fat concentrations [61]. Additionally, restricted dieters must rely on the inefficient endogenous production of EPA, and especially DHA, through the consumption of plant-based foods containing the precursor ALA [62]. The next available alternative for those adhering to preferential diets is *n*-3 LCPUFA supplementation in oil, powder, or capsule form including algal oil capsules. Because of their low cost, global applicability, and minimal to negligible side effects, it would be valuable to identify nutrients capable of treating mental health illnesses as either a complement to traditional medicines or a substitute [9,63].

Implications for future research

The current study recognises encouraging results from clinical trials [47,64] involving the supplementation of omega-3 LCPUFAs showing improvements in cognitive functioning and impulse control, necessary for decision-making and emotional regulation. Evidence shows that disproportionate levels of DHA in membrane phospholipids (due to omega-3 deficiency, or overconsumption of omega-6) disrupt molecular regulation, resulting in an increased risk of neurological disorders wherein impulsivity is a key characteristic [62]. Nutritionists and lipidologists have consistently emphasized the need to consider supplementation in diets where the consumption of fish is excluded [21,24,60].

Collaborative efforts pursuant to evidence-based, transparent, beneficent, and non-maleficent research are crucial for advancing progress in managing and treating mental health conditions [65]. The anticipated benefits of the current research include providing direct and applicable support in the common pursuit of increased practical significance of findings for future similar studies—specifically, studies that include self-report measures as part of their methodology. Furthermore, any produced benefits may likely flow to research investigating non-pharmacological treatments, such as algal omega-3 LCPUFA supplementation, to complement traditional medicines for impulsivity-related conditions or to people who follow preferential or restricted diets.

Study limitations

One limitation of this study is the lack of biomarkers. Ideally, and for greater accuracy, the use of biomarkers in research is commonly used. However, within the ethical and practical constraints of the present study, the PUFA FFQ was validated against blood biomarkers and was shown to be both reproducible and valid as a measure of omega-3 and omega-6 intakes [28,29,30].

Unsupervised, online questionnaires theoretically provide a sense of privacy for the participant and are assumed to elicit more candid responses [66]. However, this is not always the case, mainly when questions of a highly sensitive, intrusive, or morally questionable nature are presented [66,67]. The exclusionary nature of vegan and vegetarian diets often attracts disproportionate scrutiny in health research compared to non-restricted diets and is often labelled as nutrient-deficient and incongruent with mainstream health guidelines [37]. As such, some degree of defensiveness and or inaccurate reporting is possible due to the perceived bias in health research.

The validity of self-report questionnaires such as the BIS-11 and PUFA FFQ used in this study relies on the authenticity of participant responses. Accordingly, this study attempted to strike an ethical balance between research transparency and socially desirable responses by withholding the expected relationship between restrictive diets and higher impulsivity. Despite these efforts, it is plausible that vegans and vegetarians may have responded to BIS-11 questions more cautiously regarding their dietary preferences. Described as a biased or primed response, negative stigma poses a significant barrier in capturing accurate data when using self-report measures, especially among respondents who perceive any content as judgemental or a threat to their constitution [68].

A final limitation was the potential for other substances that could regulate mental health were not considered.

## 5. Conclusions

Both vegan and vegetarian groups had significantly lower omega-3 LCPUFA dietary intakes than the non-restricted dietary group, and those on the vegan diets scored significantly higher than the other groups regarding the second-order attentional aspect of self-reported impulsiveness. These findings highlight the potential relationships between dietary restrictions, lower omega-3 LCPUFA intakes and some aspects of impulsiveness, which should be investigated in subsequent larger longitudinal studies that utilise objective measures of impulsivity.

## Figures and Tables

**Table 1:** LCPUFA intakes (median intakes in grams).

	Non-Restricted Diet (*n* = 212)	Vegetarian Diet (*n* = 45)	Vegan Diet (*n* = 76)	χ2(2)	*p* Value
Total Omega-3 intakes (EPA + DPA + DHA)	0.347 ^a^	0.024 ^b^	0.000 ^c^	184	<0.001
EPA intakes	0.105 ^a^	0.006 ^b^	0.000 ^c^	182	0.001
DHA intakes	0.141 ^a^	0.013 ^b^	0.000 ^c^	166	<0.001
Total Omega-6 intakes (LA and AA)	10.70 ^a^	9.73 ^a^	16.32 ^b^	16	0.002

Different superscript letters indicate differences between individual groups with significance level *p* < 0.017.

**Table 2:** BIS-11 total scores and second-order factors (mean ± SD).

	Non-Restricted Diet (*n* = 212)	Vegetarian Diet (*n* = 45)	Vegan Diet (*n* = 76)	F (2330)	*p* Value
BIS-11 total scores	59.9 ± 9.24	60.3 ± 8.93	62.9 ± 9.7	2.931	0.055
Second order factors Attentional (Att2nd)	16.1 ± 3.87 ^a^	16.8 ± 4.20 ^a^	18.0 ± 4.41 ^b^	6.193	0.002
Motor (Mot2nd)	21.2 ± 3.76	21.0 ± 3.48	21.6 ± 3.65	0.399	0.671
Non-planning (NonPl2nd)	22.6 ± 4.51	22.5 ± 4.21	23.4 ± 4.79	0.843	0.431

Post hoc multiple comparisons using the Hochberg’s GT2 test indicated that participants following a vegan diet had significantly higher mean Att2nd scores than the non-restricted group (*p* = 0.002), 95% CI [17.96, 4.41]. Different superscript letters indicate differences between individual groups with significant level *p* < 0.017.

## Data Availability

Data are available upon reasonable request from the first author.

## Appendix A

**Table A1:** BIS-11 Items depicting first- and second-order factor loadings [30].

Item No.	Item Content	First-Order Factor	Second-Order Factor
5	I don’t “pay attention”	Attention	Attentional
9 *	I concentrate easily	Attention	Attentional
11	I “squirm” at plays and lectures	Attention	Attentional
20 *	I am a steady thinker	Attention	Attentional
28	I am restless at the theatre	Attention	Attentional
6	I have racing thoughts	Cognitive Instability	Attentional
24	I change hobbies	Cognitive Instability	Attentional
26	I often have extraneous thoughts	Cognitive Instability	Attentional
2	I do things without thinking	Motor	Motor
3	I make up my mind quickly	Motor	Motor
4	I am happy-go-lucky	Motor	Motor
17	I act “on impulse”	Motor	Motor
19	I act on the spur of the moment	Motor	Motor
22	I buy things on impulse	Motor	Motor
25	I spend or charge more than I own	Motor	Motor
16	I change jobs	Perseverance	Motor
21	I change residences	Perseverance	Motor
23	I can only think about one thing at a time	Perseverance	Motor
30 *	I am future-oriented	Perseverance	Motor
10 *	I save regularly	Cognitive Complexity	Non-Planning
15 *	I like to think about complex problems	Cognitive Complexity	Non-Planning
18	I get easily bored when solving thought problems	Cognitive Complexity	Non-Planning
27	I am more interested in the present than the future	Cognitive Complexity	Non-Planning
29 *	I like puzzles	Cognitive Complexity	Non-Planning
1 *	I plan tasks carefully	Self-Control	Non-Planning
7 *	I plan trips ahead of time	Self-Control	Non-Planning
8 *	I am self-controlled	Self-Control	Non-Planning
12 *	I am a careful thinker	Self-Control	Non-Planning
13 *	I plan for job security	Self-Control	Non-Planning
14	I say things without thinking	Self-Control	Non-Planning

Note. * indicates reverse-scored items.
